# Hierarchy and inequality in research: Navigating the challenges of research in Ghana

**DOI:** 10.1177/14687941221098927

**Published:** 2022-05-15

**Authors:** Magnus Mfoafo-M’Carthy, Jeff Grischow

**Affiliations:** 8431 Wilfrid Laurier University, Waterloo, ON, Canada

**Keywords:** Hierarchy, inequality, Ghana, culture, qualitative research, disability, mental health, caregivers

## Abstract

This paper provides insights from experiences in data gathering and recruitment from two research projects on disability/mental health in Ghana. The focus of the study explores stigma amongst individuals diagnosed with mental illness and their caregivers. The study investigates the positioning of the researcher in a superior light by participants which often wrests power from those who should be considered the true experts of their own circumstances. Inequality in the interview process thus carried the risk of impacting the quality of the data, as some participants did not consider themselves as ‘experts’ of their condition. The paper explores strategies for addressing these challenges of hierarchy and inequality in the research process in the Global South. Based on the study, we report on our experiences as follows: (1) ensuring that participants are empowered to engage with researchers; and (2) training local researchers to engage in culturally sensitive research processes.

## Introduction

Journeying from the Global North to the Global South and vice versa is often fraught with challenges with cultural norms and beliefs, language and other ways of life. In the same vein, undertaking research in the Global South is also saddled with challenges as the researcher is compelled to learn and follow cultural practices in order to accomplish an ethically sound study. Some of the challenges likely to be confronted when conducting research in the Global South include the cultural norms of a community and how these norms relate to the community being studied. Though it has been suggested that the world is a ‘global village’ due to globalization (Mfoafo-M’Carthy and Akesson, 2019), one should not lose sight of the fact that despite the easy movement of individuals from one country and culture to another, it is paramount to be conscious of and culturally sensitive to the beliefs and practices of other countries.

Over a period of more than 5 years, the authors have engaged in research projects in Ghana. From the beginning we have been concerned with designing our projects in a way that privileges the voices of Ghanaians with disabilities, who have been neglected in academic work on Ghana as well as stigmatized in Ghanaian society. In a general sense, we take an approach similar to Participatory Action Research (PAR), in which researchers work in partnership with local communities to carry out research based on the experience of the participants, that can be used to develop strategies for positive change (Baum et al., 2006). Our studies, which have involved working with individuals diagnosed with serious mental illness and other forms of disability, have sought ethics approval from the university where both authors are employed. Over the years, we have realized that the Research and Ethics Board (REB), which is tasked with reviewing such applications, tends to ask numerous questions and often requests clarification on many of the issues raised. These questions, from our perspective, are often based on a Eurocentric perspective or lack of cultural knowledge on the part of ethics reviewers (Alvares, 2011; Ker, 2013).

The focus of this study was to explore the challenges of individuals diagnosed with serious mental illness/disability and their caregivers in Ghana. For this study, 120 individuals and their caregivers were interviewed in different regions of Ghana to investigate their perception and understanding of stigma (Goffman, 1963) and how that pertains to the challenges faced by individuals living under similar conditions in Ghana. The interviews were conducted in Accra, the capital of Ghana; Kumasi, the cultural capital in the Ashanti; Cape Coast/Takoradi, in the Central and Western regions, respectively; and the Savannah regions in the northern part of the country.

In order to establish the context of our research sites, it is important to provide a brief socioeconomic and cultural overview of the country. According to Ghana Statistical Services, Ghana’s population reached 29.6 million in 2018 (2018). This shows a remarkable growth in population since independence when the population was around six million. Since the country’s colonial period prior to 1967, urban areas, which include the capital city, Accra, have customarily been supplied with amenities which have led to the rapid development of these communities in comparison to the rural areas. This development has resulted in the migration of youth and adults to urban areas in search of greener pastures. Thus, in contrast to the Savannah regions in the northern parts of the country, the urban cities tend to attract more people due the availability of jobs and the propensity for better education and modern ways of life. According to the World Bank, Ghana attained middle income country status in 2010 with the discovery of oil. The economy has steadily grown by 7% per average each year since then. Despite the steady economic growth, inequality continues to increase as poverty remains prevalent with unemployment at its zenith. The country’s inequality has been attributed to the uneven distribution of wealth, particularly among politicians who are perceived to hold the strings of the national purse. Hence, corruption has impacted the allotment of resources to those rural communities where they are much needed.

Socially, the structure of the country is hierarchical where those who hold power or are wealthy in society tend to be accorded enormous respect. In Ghana, the elderly as well as those in authority are highly respected, and most citizens who fall within this category are men. Unfortunately, in the Ghanaian society’s structure, women tend to be at the bottom of the totem pole as they are ‘neither seen nor heard’. This standard continues despite the robust legislative and policies initiated by the government to enhance the well-being of women and to ensure that women are equitably accorded their right to education, politics, sustainable development and reproductive health (Dzorgbo and Gyan, 2016).

These Ghanaian realities are significant to our research because, as we shall see below, social hierarchies, inequality and widespread poverty combine make mental health care very difficult to access for most Ghanaians suffering from mental illness. We also discovered that Ghanaian socioeconomic contexts tended to be difficult for our Research Ethics Board (REB) to understand.

## Literature review

Over the past couple of decades there has been an increased concern over studying, theorizing and redressing the inequalities and power dynamics in social sciences research (Choo and Feree, 2010). This is particularly obvious in studies undertaken by researchers from the Global North undertaking studies in the Global South. Although the majority of the scholarly discussion has tended towards the theoretical, there have been some insights with respect to praxis as well. Research ethics, for instance, could deemphasize normative, prescriptive and consequentialist trends in favour of particularist, deontological or relativist theories that are more adaptable to different social and cultural contexts. Research itself could take the form of participatory action research to foreground the agency of the research subjects as co-creators of knowledge and as vested parties. A crucial aspect concerns the researchers themselves, who through a sustained reflection on their own constituted identities and positions relative to their research subjects, must address the discursive, social and material power dynamics that are inherent in the research process. And finally, research outputs could aim to decolonize knowledge as far as possible to limit the impact of the researchers needs and norms and the imposition of a singular ‘objective’ interpretation, and to foster a view that knowledge is co-created by researcher and researched from their respective situated positions. Ultimately, it is impossible to erase inequality and hierarchy from the research process, but by better recognizing the existence and impact of those power dynamics, it may be possible to undertake more equitable and impactful research.

The natural starting point for research that aims to confront its own inherent power dynamics begins during the design phase. This includes developing an appropriate ethical framework to guide research, a research model that enhances the place of the research subject, and a sensitivity to local social and cultural realities. The first and third points can be broached by recognizing the limits or drawbacks of universalist ethical frameworks, especially those rooted in the Western Enlightenment tradition. This is especially the case for research conducted in the Global South (Godard et al., 2014; Zhang, 2017), but it is also true for research conducted by and within the Global North (Gustafson and Brunger, 2014). In either research context, the problem is rooted in the uncritical acceptance of prescriptive systems that do not account for the lived realities and local context of research subjects, or even the exigencies of the researcher’s own discipline. In the surveyed literature, warnings abound concerning the following: failing to include ‘geographically sensitive’ approaches to research and the problem of imposing medical or scientific norms on the social sciences (Zhang); imposing categories of difference and vulnerability on the community being studied (Gustafson and Brunger, 2014); ignoring social and cultural differences that exist across the globe when developing guidelines (Ali, 2015; Godard et al., 2014); and assuming that established categories of difference in one region – such as gender – are invariably applicable in another region (Carstensen-Egwuom, 2014).

For the second point – developing an appropriate model of research – the focus is often on participatory action research (PAR), which aims to bring the research subjects more actively into the research project from beginning to end whilst avoiding a situation where questions, methodology and outcomes are established without their consultation or without their interests in mind. PAR, which gains its distinctive value by making subjects agents of the research process, sees research as transformative and not just descriptive. This gives the subjects a voice and a vested interest in the research and allows them to contribute their expertise to the shape of the research and its outcomes (Gustafson and Brunger, 2014). On a theoretical level, PAR acknowledges that research knowledge is co-created and reciprocally generated, and it should benefit all involved (Ozano and Khatri, 2018). The recognition that all knowledge is situated and subjective (Rose, 1997) can redress the power dynamic between researcher and researched. This can even lead to practical changes, when it is recognized that the researcher exerts power simply by deciding the questions to ask and the flow of discourse (Rose, 1997).

Ultimately, all of these concepts aim to de-centre to some extent the researcher’s power within the research process, whether by re-evaluating the respective roles of, and dynamics between, researcher and researched in the production of knowledge, or by questioning and qualifying the cultural foundations of the researcher’s privileged position of power. And as pointed out, those same concerns can be examined throughout the research process, especially through a sustained appeal to reflexive positionality.

Through the insights of feminist scholarship, issues of reflexivity, positionality and intersectionality have a profound value to researchers, whether female or male, as they aim to recognize the hierarchies and subjectivities that exist in the research process. Firstly, positionality and intersectionality provide the theoretical framework to define categories of difference, and to define the contingent and composite nature of identity and unequal relationships (Ali, 2015; Carstensen-Egwuom, 2014). Intersectionality, first theorized in reference to black women in America (and the race-gender-class triad), has now adopted and been adapted to a wide range of contexts to theorize identity and its relationship to oppression. Positionality, moreover, contributes to the idea that knowledge and identities are situated, that they are shaped by place and context, and that relationships, though unequal, are dynamic (Mikkonen and Laitinen, 2017; Ozano and Khatri, 2018; Suffla et al., 2015). This is an important consideration – and one rooted in the work of feminist human geographers (Sharp, 2007) – to ground identities and inequalities in lived experience and environment. After all, if categories of difference are, on the one hand, discursive and socially constructed, they are also rooted in material inequalities, and to ignore that is to remain dangerously aloof from the lived realities of research subjects.

For these reasons, both intersectionality and positionality can allow for a richer or open understanding of the researcher, the research subjects and the dynamics between them, especially through the practice of reflexivity. For many social science researchers (Ali, 2015; Carstensen-Egwuom, 2014; Ozano and Khatri, 2018; Rose, 1997; Suffla et al., 2015), reflexivity is what allows for the inequalities embedded in the research process to be recognized and, to an extent, redressed. For reflexivity to be effective, however, it needs to be more than merely declarative and momentary (i.e. a brief ‘I am a white middle-class man conducting research on...or I am a Black middle-class researcher’) before setting into an otherwise unaffected research plan. Rather, it needs to be concerted and continuous, considering not just the various positions within a research relationship but also the consequences of those dynamics. This can help to shed light on ‘relations of dominance’ and ‘blind spots of power’ (Carstensen-Egwuom, 2014) in field research, highlight the complex and hybrid insider/outsider role that many researchers and research assistants can inhabit (Ali, 2015; Mikkonen and Laitinen, 2017; Ozano and Khatri, 2018; Suffla et al., 2015; Zhang, 2017), and help to better situate and de-centre the production of knowledge (Ali, 2015; Mikkonen and Laitinen, 2017; Rose, 1997; Suffla et al., 2015).

It is especially in an understanding of how knowledge is produced that reflexivity can prove useful, and more than just ‘self-indulgent’ (Ali, 2015) or navel-gazing. Indeed, one of the difficulties is employing reflexivity in a way that makes a practical difference (Rose, 1997), and doing so whilst recognizing that not all inequalities can be overcome (Moss, 1995). On the one hand, positionality and reflexivity, by better recognizing the agency of research subjects and their own situated knowledge, can lead to more transparent research methods and a positive reshaping of fieldwork relationships, and ultimately to useful and empowering knowledge that has been created in a non-exploitative manner (Ali, 2015). On the other hand, combined with an appropriate methodology, greater trust between researcher and researched can also be established (Zhang, 2017). All of this points to the potential for wide-ranging change, including changes in research outcomes. There is the recognized need not only to foreground the role of research subject in the production of knowledge, and to develop a methodological reflexivity (Carstensen-Egwuom, 2014), but also to find a way, afterwards, to portray the voices of research subjects without colonization or domination (Suffla et al., 2015). This includes avoiding the ‘God trick’ (Carstensen-Egwuom, 2014, and many others) of an omniscient authorial voice of the researcher, instead prioritizing the subjective over the objective (Moss, 1995).

As Ali points out, reflexive positionality will not solve all the problems; however, ignoring its insights can be even worse (2015). And when tied to a research framework that is sensitive to local cultural expectations and social needs, there is the potential for a robust approach toward research that acknowledges the deep inequalities that exist in research, both those social inequalities imported from the wider world, and those inequalities that are unique to the research itself – especially since social inequality, according to Mikkonen and Laitinen (2017), can lead to epistemological inequality. There is a need for research frameworks that are not rooted exclusively in the cultural norms and expectations of the Global North, and for research fieldwork that self-consciously seeks to minimize (or at least recognize) the hierarchies that place the researcher in a privileged position of power. However, old binaries should not simply be replaced by new binaries. Rather, reflexive positionality shows that power dynamics, identities and indeed knowledge are situated, subjective, contingent and rooted in material existence. Thus, research practices and the ethical principles that guide them need to be responsive to that reality. Though inequalities in the research process will not be overcome simply by invoking reflexivity, the power relations that inform inequalities can be made more transparent. As well, knowledge can be more appropriately situated and decolonized to some extent.

## Research design and initial ethics review

Our experience with cross-cultural research arises from two projects on disability in Ghana, both funded by the Social Sciences Research and Humanities Council of Canada (SSHRC). The first was a 2-year program on stigma and disability, with a major focus on psychosocial disabilities, carried out between 2017 and 2019. The second is a 4-year program on the history and lived experience of disability rights, which also began in 2017. We have completed the data collection phases of both projects, during which we interviewed 120 participants for the stigma/psychosocial disability project and 300 participants for the history/lived experience project. In both cases, we developed the initial research design using standard Western methodological approaches in order to satisfy the requirements of our institution’s Research Ethics Board (REB) and to act in accordance with the ethical guidelines provided by the Tri-Council (the parent body overseeing SSHRC funding as well as funding from the Canadian Institute of Health Research and the National Science and Engineering Council of Canada).

To this end, we developed semi-structured questionnaires that included questions about the participants’ lived experience with stigma and, for the oral history project, experiences with membership and/or leadership in Ghana’s national disability associations. The questionnaires, developed by the lead researcher, posed questions on the participants’ backgrounds as well as their experiences with disability including stigma, hospital treatments, economic consequences, and, in the case of the oral history project, leadership experience in disability associations. To recruit participants, we proposed using a combination of purposive and snowball sampling. We set out to interview disabled Ghanaians who represent a subset of the larger population. However, their total numbers are not well known, especially if we divide them according to disability types. Accessing the target population is also difficult because of the lack of certain infrastructural resources such as street names. For this reason, we began by using purposive sampling. Firstly, we asked our existing contacts within the Ghanaian disability community to identify the first pool of interview subjects. After this, we utilized snowball sampling through which our contacts and participants identified additional interviewees through word-of-mouth. We decided to offer compensation to the participants because we believed that it was ethically important since we were working in Ghana where poverty levels are very high.

At the start of our projects the REB raised several minor issues before granting ethics approval, none of which were particularly tailored to Ghanaian contexts. For the stigma/psychosocial disability project, they asked us to clarify whether the informed consent and interviews would be delivered through different methods for different disability groups (e.g. read aloud to blind participants or administered by sign language to deaf participants). We were also asked to designate specific amounts for compensation, clarify that participants could deny recording of interviews at any time, and clarify that all data would remain confidential. For the oral history/lived experience project, the REB simply asked us to ensure that we trained all interpreters, protected the participants’ confidentiality and obtained consent for publishing direct quotations (but with pseudonyms to protect identities).

## Engaging in research in Ghana

With our ethics approvals in place, we began our fieldwork for both projects in late 2017 and 2018. Once in the field, we encountered conditions and circumstances that challenged some of the standard Western notions of research design and ethical fieldwork. Data gathering for these studies entailed using various approaches in the recruitment process. These included snowball, purposive and other recruitment samplings. Our project on psychosocial disabilities provides an illustrative case. At the psychiatric hospital in Accra, for instance nurses in the different units assisted with the recruitment process by recommending individuals deemed capable of participating in the study. We had to meet with these individuals for their consent. The question from the ethics board that later came to light was whether these patients voluntarily agreed to participate or felt coerced by the nurses, who were in charge of the different units. Did the participants agree to participate because they had a story to tell? If so, did they feel confident about the fact that they owned their stories and it was their voices that were being sought? The researchers at one point felt differently than they had at the onset of the study, as most of the participants’ body language and nervous tone conveyed discomfort with the interviews. This made us wonder whether the participants felt that they could be honest in their responses, for example when given opportunities to the hospital and its staff knowing very well that they would have to interact with them after the interviews (Author, in Press). Participant discomfort over sensitive questions has been an issue in fieldwork for a very long time, including research in Ghana such as Bleek’s work in the 1970s that revealed systematic lying by informants. We wondered about this possibility as well, but two factors lead us to believe in the veracity of our data. Firstly, the research team in this case was entirely Ghanaian, meaning that the interviewers themselves had a deep understanding of the social and cultural context. Secondly, our data analysis showed similarities in the issues raised by the informants, despite the fact that they did not know each other and had no opportunities for collaboration.

In the process of gathering the data in the other regions, recruitment of participants was challenging as the researchers had difficulty selecting participants. In some cases, the participants did not feel confident sharing their personal stories. On a number of occasions, they tended to ask whether what they are sharing was what the researcher wanted. They often asked questions like ‘Is that what you want?’ or ‘Is my story in line with what you are looking for?’ Despite assurances that it is their experience and they should feel comfortable telling us what they have experienced, they kept asking such questions. Also, they tended to put the researchers on a pedestal by being at our beck and call and ensuring that we were happy with the process. The question we kept asking is whether the reception would have been the same had the researchers been locals.

The caregivers in all the communities we visited were more than willing to participate, which we appreciated. However, there were occasions when caregivers would bring other family members and introduce them as also willing to participate in the study because they were also taking care of family members or had taken care of a disabled family member in the past. Probing further eventually revealed that the caregivers assumed we had ample money to give out and were also coming for their share of the money being distributed. This tendency made us reflect deeply on our positionality as researchers entering Ghana as part of a project funded by institutions in the global North. We did indeed have ample funds, but we had to disburse them in culturally appropriate ways so that our respondents were not overly compensated and motivated to participate mainly by financial gain. On another matter, the caregivers’ responses, like those of the participants, also showed they were willing to say what they felt the researchers wanted to hear. This was despite the researchers’ assurances that it was the participants’ story and they owned it. This painted a picture of inequality as we were deemed superior and placed on a pedestal as they tried to please us for the remuneration. We wondered whether this was due to us being foreign researchers.

A second component of our research investigated the lived experience of Ghanaians with physical disabilities in a cluster of small towns in Ghana’s Eastern Region. We connected with this community through a contact who is the head of an informal disability association. The interviews were conducted by a Ghanaian graduate student, who was completing a degree in disability studies. This graduate student was assisted by another contact who is a long-time resident of the main project site and disabled himself. Soon after we began interviewing our participants, a number of cross-cultural issues came to light. Firstly, most of the participants assumed that we (the Canadian researchers) were conducting the interviews in order to bring financial assistance from Canada. Secondly, word quickly spread that we were offering honoraria for participating, which prompted many additional people to come forward who were not on the original list of participants. Thirdly, we soon realized that the interview questions were not eliciting sufficient data with respect to the personal stories of the informants and their experiences with disability within local social and cultural contexts. These issues prompted us (researchers) to think deeply about our positionality as researchers from the Global North entering a community in the Global South. Seeking not to impose Western research paradigms on our participants, we turned to our Ghanaian assistants for guidance as cultural brokers (more of which below). Most importantly, they worked together to explain to the participants that we were interested in collecting life stories in order to build an understanding of the experience of disability in Ghana. Rather than bringing money from outside, their stories would be shared globally in the interests of advocacy. With respect to the would-be participants coming to us when they heard about the payments, our main local contact was able to explain to them that we were conducting a project within a limited timeframe and budget, and that we would not be able to interview everyone. As researchers based in Canada, this experience was one of the most difficult aspects of the project personally. As a partial solution, after discussion with our local contact, we decided to hold a group meeting during a subsequent visit to allow more people to share their stories. A church offered their space for the meeting, we provided drinks and snacks, and the result was a lively and informative discussion with close to 50 participants who were mostly either physically disabled or blind. Because we relied on a local contact (himself disabled) who served as a cultural broker, we were confident that the venue and the drinks/snacks were the most appropriate arrangements to ensure that the participants were comfortable with the proceedings. This proved correct, and many of the invitees freely shared their stories.

Interviewing deaf participants proved especially challenging. By working through a deaf contact in the study location, we were able to find 20 participants. The research assistant was trained in Ghanaian Sign Language (GSL). However, when they got to the field and began the interviews, they realized quickly that many of the participants had not progressed beyond the most basic level of education and therefore were not fluent in GSL. Simply targeting a population by disability type therefore was not an adequate recruitment method; the pool of participants had to be narrowed to include only those with sufficient training in GSL to communicate clearly and fully. This raised an ethical issue that cannot be fully resolved, because out of necessity we were forced to remove the least educated Ghanaians with deafness from our study population, even though they likely might have as much or more to say about stigma and discrimination. This problem was brought home in another location, where we attempted to interview participants with poor sign language skills (none in some cases) and came away with very little useable data. It is a problem that even our deaf Ghanaian colleague have not been able to solve.

For the oral history interviews, we wanted to recruit a large pool of participants who had worked in Ghana’s disability associations from the 1960s to the present. We relied on Ghanaian contacts who are disabled themselves and who have connections to the Ghana Blind Union (GBU), Ghana Society for the Physically Disabled (GSPD), and the Ghana National Association for the Deaf (GNAD). Able to recruit more than 200 respondents, their positionality as disabled Ghanaian researchers helped greatly to establish trust and ensure that the interviews were conducted ethically with the full consent of the participants. These participants, the most highly educated of our respondents, had served in professional careers. Their social and economic status meant that they did not expect that we would bring financial assistance from abroad. However, we decided to increase their remuneration for the interviews to 100 cedis (Ghanaian currency). We made this decision after consulting our Ghanaian contacts/cultural brokers, who recommended higher payments to the participants who had a higher social standing. In Ghanaian culture, it is important to show respect to seniors/elders, and it is common practice for researchers to provide larger payments for participants with a higher social standing and better financial situation. This is unlike Western research practice where remuneration is not necessarily based or linked to the participants’ social standing. It should also be mentioned, however, that the oral history participants lived in larger urban centres than the others and that their interviews lasted longer. The need to modify our methodology precipitated a lengthy exchange with our REB over ethics in Ghanaian research, especially with respect to payments to participants. Initially the REB rejected our plan to pay seniors/elders more than others for participating in the interviews. Instead, they asked us to choose an amount between 30 and 100 cedis (equivalent of 10 and 35 Canadian dollars) for all participants, regardless of social rank or location. There ensued a series of exchanges over a 2-month period with the REB over the compensation issue, during which time we were forced to suspend our work in Ghana. After multiple e-mails, we finally obtained the REB’s approval and were able to resume our fieldwork.

The issues raised by our REB did not reflect ill will or biases against non-Western research, but rather the board members’ lack of experience with research in Africa, and Ghana specifically. This lack of experience led the Board to question the ethics of the project as they worked to interpret the Tri-Council guidelines. In our opinion, the guidelines themselves are not the problem as they are broad enough to fit cross-cultural contexts. For example the Tri-Council policy on compensation states that incentives ‘should not be so large or attractive as to encourage reckless disregard of risks’, or to encourage recruits to participate in order to ‘gain favour or improve their situation’. In setting appropriate amounts, ‘researchers and REBs should be sensitive to issues such as the economic circumstances of those in the pool of prospective participants, the age and decision-making capacity of participants, the customs and practices of the community, and the magnitude and probability of harms’ (TCPS 2, p. 29). In our case, we were guided mainly by community customs, including social rank, as well as different economic circumstances in urban and semi-rural areas. It was difficult to explain these considerations to our REB but eventually we succeeded on the issue of compensation.

## Discussion and conclusion

Based on our experiences, we have come to realize that being reflective in embarking on research in developing countries like Ghana requires being sensitive to the culture and the way of life of those being researched. There are many questions that come to mind, including What is the focus of the study? How are we expected to engage in the studies? Do we (Western-trained) researchers go in as the experts? How do we engage the services of locals? At which stage of the research, that is the data gathering stage, do foreign trained researchers come in?

Through our fieldwork experiences, we have come to realize the importance and necessity of engaging the services of locals, including gatekeepers/cultural brokers who can help with recruitment and facilitate our entry into Ghanaian communities. Although gatekeepers and cultural brokers can complicate and sometimes undermine fieldwork, they also ‘can be helpful facilitators who provide access to and increase acceptance among research subjects and who help interpret cultural/political issues’ (Campbell et al., 2006: 103). We certainly found this to be the case in Ghana, especially after providing our local contacts with the training needed to assist with the study. Training locals can be challenging due to the nuances associated with qualitative research, but they are the gatekeepers of traditional culture and thus the most capable of addressing the norms and challenges that come with it. They are the faces the participants see and are used to, they speak their language and communicate well, and they are also cognizant of the mannerisms of the participants. In many ways, our local cultural brokers resembled Meta Carstarphen’s ‘transfluencers’: local ‘who can both translate (literally and figuratively) the cultural experiences before us, as well as influentially create a more intimate kind of access’ (Carstarphen, 2014: 178). Working through our local contacts, we sought to engage the participants in a way that minimized signs of ‘superiority’ of the researchers. We also realized that remuneration for research should not be based on the expectation of Western institutions but on what is deemed culturally appropriate. In some cultures, it is considered appropriate to provide food or gifts and not money. Still, we remain cognizant of the ongoing challenge of ensuring that the perception of elevating Western researchers as wealthy donors is challenged. Also, we believe very strongly that REBs must be educated about and sensitized to research in different communities. The ‘one-size-fits-all’ mentality adopted by REBs can be detrimental to studies abroad, particularly in countries where cultural norms from those in the Western world.

One can argue that in exploring these issues, it is not so much about positionality or social location as foreign researchers, but more importantly about locating our own privileged path and how that influences our work. Choules (2007) states that,

We need to scrutinize our own assumptions, beliefs, and behaviours and question the normalized position from which those beliefs grow. Unless we do that and engage in action to undo privilege, those of us in privileged positions are implicated in the injustice created. (p. 478)

The fact is that as researchers, we do not want to be implicated by or complicit to an unjust or unequal system that pits the haves against the have-nots. Hence, we are called to use our positions to be reflective and to use our voice to challenge the status quo and work toward the implementation of a system that is fair, irrespective of one’s positionality.

We therefore recommend the involvement of local researchers in research projects either in the form of research assistants or gatekeepers. These individuals would be familiar with the community, and they would play a significant role in the recruitment process. These individuals could be trained and equipped to engage in the recruitment and interview process.

This paper explores the challenges associated with navigating research projects in developing countries such as Ghana. Some of the challenges the authors have encountered over the years in the process of data gathering are discussed, along with how these challenges affect the quality of the study being undertaken. Some of the main challenges pertain to the cultural beliefs and the sociological dimension of the society where the research is being conducted. For instance Ghana is culturally a hierarchical society where those with power, including money, are literally celebrated. Thus, being seen as a researcher from a Western country connotes some form of superiority, which tends to undermine the study being conducted.

Due to cultural differences and the different ways of life of individuals in different countries, it is important for researchers to be culturally sensitive and willing to critically evaluate their stance and approach toward the subject under study. This sensitivity is not only applicable to the researchers but also to the research review boards which tend to use the optics of research practices in the global North as the yardstick for all forms of research. It is now timely for education to bridge the gap between research in the North and South dichotomy.

It is therefore recommended that local researchers be employed and engaged as gatekeepers and research assistants to guide and educate Western researchers on the norms and approaches of community-based research. This will ensure that local researchers will be able to engage in culturally sensitive research processes in the country and also, research participants will be empowered to confidently engage with researchers.
